# Effects of neuromuscular lags on controlling contact transitions

**DOI:** 10.1098/rsta.2008.0261

**Published:** 2009-02-16

**Authors:** Madhusudhan Venkadesan, Francisco J. Valero-Cuevas

**Affiliations:** 1 Department of Mathematics, Cornell UniversityIthaca, NY 14853, USA; 2 Department of Biomedical Engineering, University of Southern CaliforniaLos Angeles, CA 90089, USA; 3 Division of Biokinesiology & Physical Therapy, University of Southern CaliforniaLos Angeles, CA 90089, USA

**Keywords:** contact transition, time lag, finger, hand, speed–accuracy

## Abstract

We present a numerical exploration of contact transitions with the fingertip. When picking up objects our fingertips must make contact at specific locations, and—upon contact—maintain posture while producing well-directed force vectors. However, the joint torques for moving the fingertip towards a surface (***τ***_m_) are different from those for producing static force vectors (***τ***_f_). We previously described the neural control of such abrupt transitions in humans, and found that unavoidable errors arise because sensorimotor time delays and lags prevent an instantaneous switch between different torques. Here, we use numerical optimization on a finger model to reveal physical bounds for controlling such rapid contact transitions. Resembling human data, it is necessary to anticipatorily switch joint torques to ***τ***_f_ at about 30 ms before contact to minimize the initial misdirection of the fingertip force vector. This anticipatory strategy arises in our deterministic model from neuromuscular lags, and not from optimizing for robustness to noise/uncertainties. Importantly, the optimal solution also leads to a trade-off between the speed of force magnitude increase versus the accuracy of initial force direction. This is an alternative to prevailing theories that propose multiplicative noise in muscles as the driver of speed–accuracy trade-offs. We instead find that the speed–accuracy trade-off arises solely from neuromuscular lags. Finally, because our model intentionally uses idealized assumptions, its agreement with human data suggests that the biological system is controlled in a way that approaches the physical boundaries of performance.

## 1. Introduction

Every day we use our fingertips to make and break contact with objects as we interact with them. This often requires abruptly transitioning from moving the fingertip towards a specific location on the object to producing well-directed force vectors. We recently found (Venkadesan & Valero-Cuevas 2008) that the human nervous system achieves this via an anticipatory and neurally demanding strategy of switching muscle activations—and therefore finger joint torques—before contact (i.e. from those controlling finger motion to those controlling fingertip force). We (Venkadesan & Valero-Cuevas 2008) and many others (Hogan 1985; Whitney 1987) have demonstrated that such a strategy is sensitive to time delays and lags in the combined controller plus finger system, and to errors in planning. These observations raise several questions: Does the strategy used by the biological system for contact transitions approach a strategy that is mechanically optimal or near optimal; or is it driven by biological considerations/limitations that are not typically included in biomechanical models? In addition, given that the rapidity of the task precludes the benefit of sensory feedback, how sophisticated and accurate does the motor programme need to be? What are the limits to the mechanical performance of the task given the inherent dynamics of muscle function? As a first step towards answering these questions, we tested whether an open-loop optimal controller for an ideal mathematical model of the finger (i.e. a planar, frictionless, torque-driven mechanical system with free hinge joints) would transition between joint torques in a manner that resembles electromyographic (EMG) measurements in humans.

## 2. Experimental findings on human finger contact transitions

**Figure 1:**
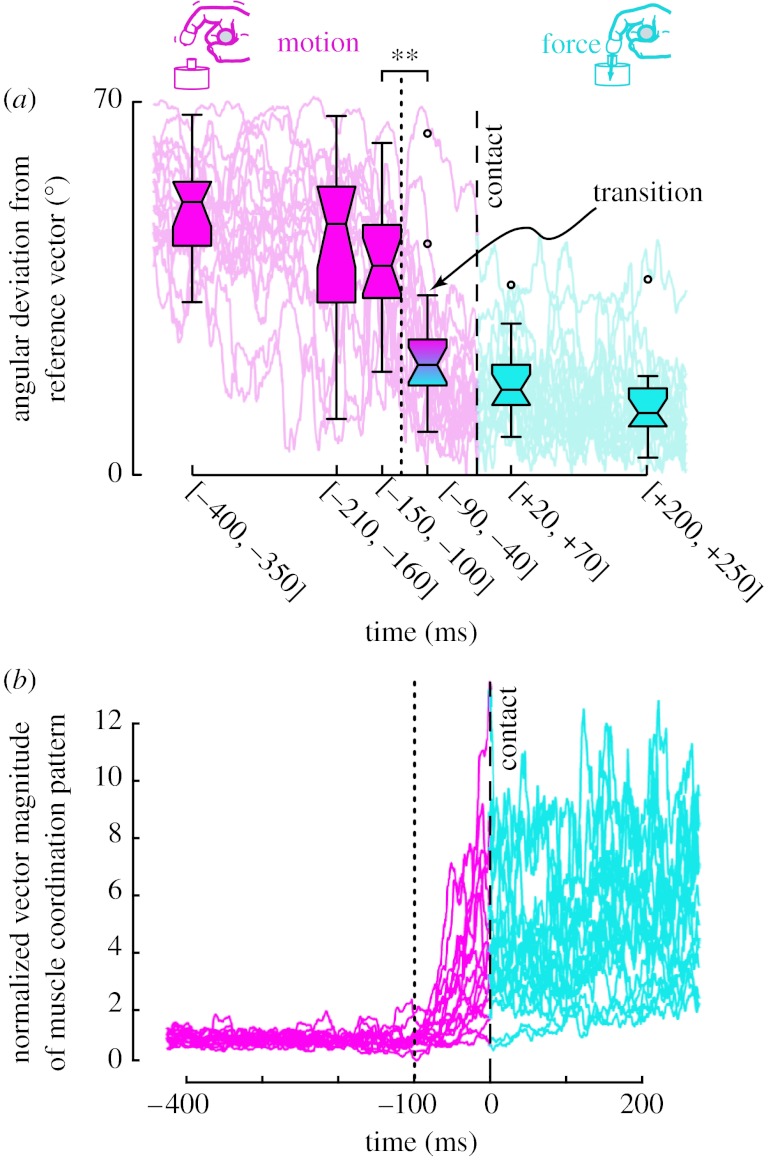
The main experimental findings of our prior work. Adapted from Venkadesan & Valero-Cuevas (2008). (*a*) The seven EMG signals were assembled into a seven-dimensional EMG vector weighted by the relative strength of the muscles. To track the temporal change in direction and magnitude of the muscle coordination vector, we used (i) the scalar angle between the instantaneous EMG and a reference EMG vector (from static force production 500 ms after contact) and (ii) its Euclidean norm. The data show that the change in vector direction is almost complete by approximately 65 ms before contact (*t*=0). (*b*) The magnitude of the EMG vector, however, begins to increase at approximately 70 ms before contact occurs and continues to increase after contact. The EMG vector reflects the vector of joint torques because the EMG vector is related to net joint torques through an affine map as described previously (Valero-Cuevas *et al*. 1998; Valero-Cuevas 2006).

In a prior study (Venkadesan & Valero-Cuevas 2008), we investigated the neural control of finger musculature when the index fingertip abruptly transitions from motion to static force production. Human subjects produced a downward tapping motion followed by vertical fingertip force against a rigid surface. By simultaneously recording the three-dimensional fingertip force and EMG from all seven index finger muscles, we found that the muscle coordination pattern clearly switched from that for motion to that for isometric force at approximately 65 ms before contact (figure 1). We then used mathematical modelling and analysis to find that the underlying neural control was predictive and switched between mutually incompatible strategies in a time-critical manner. Importantly, this abrupt switch in underlying neural control polluted fingertip force vector direction beyond what is explained by muscle activation–contraction dynamics and neuromuscular noise. Therefore, we proposed that, because the neuromuscular system cannot switch between control strategies instantaneously or exactly, there arise physical limits to the accuracy of force production upon contact.

However, the prior modelling work does not establish the bounds on performance of this transition, nor does it provide any insights into the necessity for an anticipatory strategy.

## 3. Methods

### (a) Problem statement

**Figure 2:**
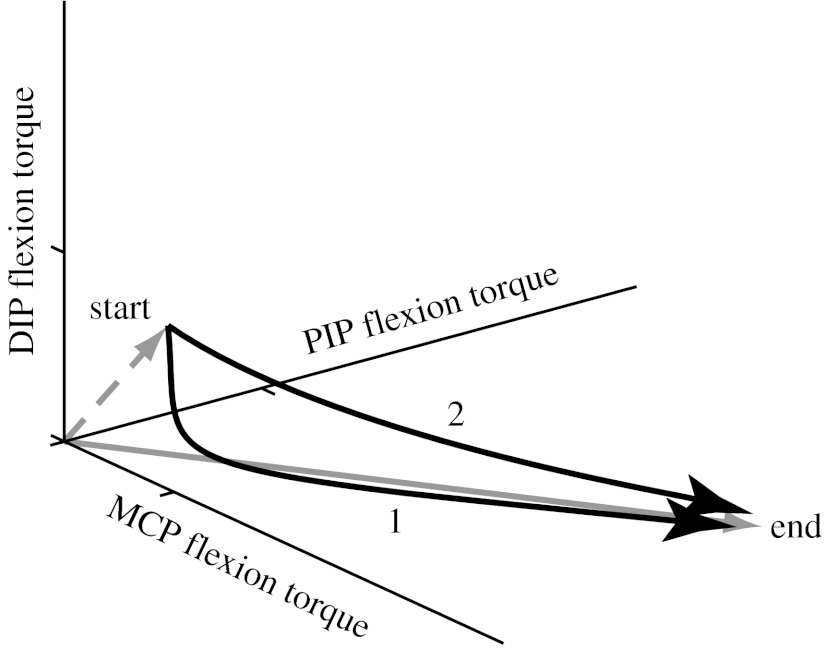
Multiple paths for transitioning from the control of fingertip motion to fingertip force. There are multiple possible paths in three-dimensional space of joint torques to transition from the torques for motion (start) to the production of well-directed forces upon contact (end). Because the neurophysiological system can transition neither instantaneously nor exactly, it must be done by interpolating in one of several ways in an open-loop manner (because the transition is faster than the delays in sensory feedback). Some possible strategies are: (1) first rotating the vector and then growing its magnitude (as in humans; figure 1) or (2) simultaneously changing both vector direction and magnitude. In this work, we find the optimal strategy so that the fingertip force vector is closest to vertical upon contact.

We seek to find an optimal open-loop control strategy for transitioning from finger motion to static fingertip force production. In the three-dimensional space of joint torques (where each axis represents the torque about one joint), this problem can be described graphically as the need to smoothly transform one vector for fingertip motion into another vector for fingertip force within a finite time (figure 2). The task goal at the specified contact time is to arrive at the surface (i) as close to the coordinates of the target location as possible, (ii) with minimal horizontal and angular velocities of the fingertip, (iii) with joint torques that produce a prescribed static force vector (magnitude and direction) upon contact (i.e. ***τ***=***τ***_f_), and (iv) with the time derivative of the joint torque vector aligned with $\tau_{\text{f}}(\hat{\dot{\tau }}={\hat{\tau }}_{\text{f}})$, i.e. the fingertip force vector should only be growing in magnitude and not changing in direction. Therefore, the specific path of the torque vector, its temporal evolution and the time at which the transition is completed are all free to change. We used numerical optimization to find the optimal control strategy that best achieves this task goal. Details of the numerical optimization are given in §3*e*.

*Notation*. Throughout the text, we use bold-italicized symbols (e.g. ***f***, ***τ***, ***N***) for vectors, roman capitals for matrices (e.g. M, C), and italicized symbols for scalars (e.g. *t*, *β*, *T*_act_). For denoting specific components of vectors, we use subscript indices; and when there is no room for subscripts, we use superscripted indices within parentheses $\left(\text{e.g.}\;\tau_{1},\tau_{\text{D}}^{\left(i\right)}\right)$. Hatted symbols denote the unit vector corresponding to the vector denoted by the hat-less version of the same symbol (e.g. $\hat{\tau }$ is the unit vector of ***τ***).

### (b) Modelling assumptions

We list below the main assumptions in building the finger model and briefly state the rationale underlying each assumption.

*Ideal finger joints*. The finger is modelled as a planar three-joint mechanism with ideal, frictionless hinge joints with no stiffness or damping. We make this assumption primarily because we want to find the neural contributions to the experimentally observed joint torque trajectories separate from passive musculoskeletal viscoelastic elements, such as ligaments, skin, etc.

*Torque-actuated joints*. Our model has joints actuated by torque generators instead of muscles. This permits us to simplify the model while at the same time to identify the physical bounds on performance of the biological system.

*Open-loop switching strategy*. We consider only open-loop control strategies in a transition time window between −60 and 0 ms. This is because we assume that physiological time delays preclude the use of sensory information in guiding the control switching during this window. Typical values for sensory time delays in finger manipulation tasks range from 65 to 120 ms (Venkadesan *et al*. 2007 and references therein).

*Torque actuator's activation and contraction dynamics*. We prescribe that activation–contraction dynamics prevent the muscle forces, and in turn the joint torques, from switching instantaneously between those for finger motion and those for static fingertip force. In the absence of this assumption, there is a trivial and physiologically unrealistic solution to our optimal control problem. Namely, the joint torques could switch instantaneously at the exact time of contact and produce a perfectly vertical initial fingertip force vector. However, a key and unavoidable limitation in the biological system is the presence of neuromuscular lags that limit the rate of change of muscle forces (Zajac 1989), and hence of joint torques.

*Collision law*. We assume that the fingertip is well damped so that high impact forces, rebound, etc. are of no concern. Additionally, we assume that damping from the fingertip and finger joints can completely dissipate the vertical and (small) horizontal velocity components of the fingertip, but not the rotational velocity. This is justifiable by the known finger pulp properties in humans (Hajian & Howe 1997; Pawluk & Howe 1999*a*,*b*; Jindrich *et al*. 2003) and our own kinematic measurements (Venkadesan & Valero-Cuevas 2008).

*Post-contact force production*. We only consider the instant of time immediately after contact to calculate the fingertip force vector (mis)direction that needs to be within the friction cone. Subsequently, of course, passive viscoelastic elements in the joints (that we have omitted in our model) and the nervous system will stabilize the force direction and finger posture. Those subsequent adjustments are beyond the scope of this work.

*Time window of interest*. We restrict the time interval of all simulations to [−60 ms, 0 ms], where the contact occurs at 0 ms. This assumption reflects our experimental observation that humans performed an anticipatory transition in EMGs. This assumption therefore restricts the class of optimal solutions that we search to anticipatory strategies. However, it does not specify how much sooner than contact the actual transition itself should occur. Our results (§4*a*) reveal some surprising findings in this regard.

### (c) Dynamic model of the index finger

**Figure 3:**
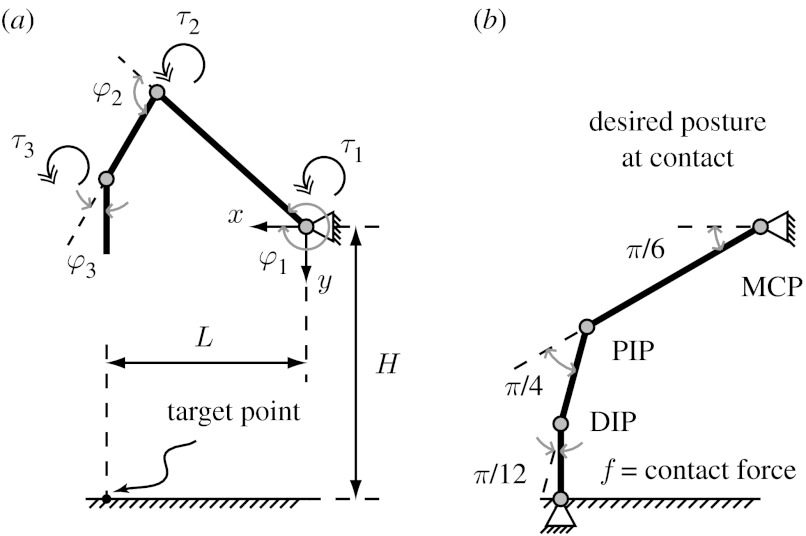
Schematic of a three-link open-chain and a four-link closed-chain model of the index finger. (*a*) Three-link model for the motion phase, which shows the posture 300 ms before contact. Joint angles *φ*_*i*_ are positive anticlockwise as measured from the positive *x*-axis. Joint torques (*τ*_*i*_) also follow the same convention. *L* and *H* are the horizontal and vertical distances to the target point from the origin of the coordinate system located at the MCP joint. (*b*) Four-link model of force production when the fingertip is in contact with the surface (*t*=0 ms). The figure shows a specific posture, namely the desired (goal) posture for force production. This posture was identical to the instruction given during our experiments. Here ***f*** is the contact force at the fingertip. Because the fingertip cannot resist any torques at the tip, we treat the contact as a frictionless joint.

We use a three degree-of-freedom planar and hinged serial mechanism driven by torques at each joint for modelling the index finger (figure 3). The numbering system we use for all variables associated with each joint is as follows: 1 for the metacarpophalangeal (MCP) joint; 2 for the proximal interphalangeal (PIP) joint; and 3 for the distal interphalangeal (DIP) joint (figure 3). The index finger model consists of three parts—finger motion, collision law and fingertip force production.

#### (i) Three-link open-chain model of finger motion

The equations of motion of a planar torque-driven three-link open-chain model were derived using a Lagrangian formulation. This is a very standard derivation and can be found in many textbooks (e.g. Spong & Vidyasagar 1989). The final equations of motion are

(3.1) $$\text{M}(\varphi (t))\ddot{\varphi }(t)+\text{C}(\varphi (t),\dot{\varphi }(t))\dot{\varphi }(t)+N(\varphi (t))=\tau (t).$$

Henceforth, all time dependences will be suppressed for the sake of readability. M(***φ***) is the symmetric (and positive definite) 3×3 inertia matrix such that the total kinetic energy of the system can be calculated as $(1/2){\dot{\varphi }}^{\text{T}}M(\varphi )\dot{\varphi }$. Then, the 3×3 matrix $\text{C}(\varphi ,\dot{\varphi })$ that contains centrifugal and Coriolis contributions to joint torques is calculated using the formula

(3.2) $${\text{C}}_{ij}(\varphi ,\dot{\varphi })=\frac{1}{2}\sum_{k=1}^{3}\left(\frac{\partial {\text{M}}_{ij}}{\partial \varphi_{k}}+\frac{\partial {\text{M}}_{ki}}{\partial \varphi_{j}}-\frac{\partial {\text{M}}_{jk}}{\partial \varphi_{i}}\right){\dot{\varphi }}_{k}.$$

Finally, ***N***(***φ***) is simply the 3×1 vector of joint torques induced by gravity.

*Numerical values for model parameters*. We assumed all phalanges to be of uniform circular cross section with a diameter of 13 mm. Because fingers have low fat content, we assumed them to be denser than average human body density, which is approximately 1100 kg m^−3^ (Behnke *et al*. 1942), and used a value of 1250 kg m^−3^. Using these, we calculated the masses and planar moments of inertia assuming each phalanx to be a cylindrical prism with its axis in the simulation plane. The lengths we used for each of the phalanges are *l*_1_=0.0508 m, *l*_2_=0.0254 m and *l*_3_=0.01905 m. All results we report were robust to biologically reasonable variations of these numbers. However, we do not present the results of that sensitivity analysis.

#### (ii) Collision law for the fingertip

The vector ***r*** comprises endpoint position (*r*_*x*_, *r*_*y*_) and its orientation, i.e. the angle relative to the vertical (*r*_*α*_). Then, the fingertip linear and angular velocities $\left(\dot{r}\right)$ are related to joint angular velocities $\left(\dot{\varphi }\right)$ through the 3×3 posture-dependent manipulator Jacobian (A(***φ***)). If the manipulator Jacobian is full rank, as in this case, the collision law specified in terms of the fingertip velocity can be rewritten in terms of joint angular velocities:

(3.3) $$\left(\begin{array}{c} {\dot{r}}_{x}\\ {\dot{r}}_{y}\\ {\dot{r}}_{\alpha } \end{array}\right)=\text{A}(\varphi )\left(\begin{array}{c} {\dot{\varphi }}_{1}\\ {\dot{\varphi }}_{2}\\ {\dot{\varphi }}_{3} \end{array}\right),$$

(3.4) $$g:\left(\begin{array}{c} {\dot{r}}_{x}\\ {\dot{r}}_{y}\\ {\dot{r}}_{\alpha } \end{array}\right)\mapsto \left(\begin{array}{c} 0\\ 0\\ {\dot{r}}_{\alpha } \end{array}\right).$$

#### (iii) Four-link closed-chain model of fingertip force production

The equations of motion for the non-slipping finger where the fingertip is in contact with the surface differ from that for free motion because of joint torques induced by the fingertip contact force. This force was calculated using Lagrange multipliers and the constraint equations for the fingertip. The resulting equations of motion are

(3.5) $$\text{M}(\varphi )\ddot{\varphi }+\text{C}(\varphi ,\dot{\varphi })\dot{\varphi }+N(\varphi )+A{\left(\varphi \right)}^{\text{T}}f=\tau$$

and

(3.6) $$f={\left({\text{AM}}^{-1}{\text{A}}^{\text{T}}\right)}^{-1}({\text{AM}}^{-1}(\tau -\text{C}\dot{\varphi }-N)+\dot{\text{A}}\dot{\varphi }).$$

Note that, to calculate the fingertip force at the instant of contact, given the finger's state just prior to contact, we only need to transform the pre-contact finger state according to the collision law (equation (3.4)) and then evaluate the algebraic equation for the contact force (equation (3.6)).

### (d) Joint torque model with ‘activation*–*contraction’ dynamics

We do not explicitly include muscles or other skeletal structures, such as tendons, ligaments, skin, etc., in our model. As discussed earlier (§3*b*), we want to avoid trivial solutions without imposing arbitrary constraints on the evolution of joint torques prior to contact. We therefore use a model for the torque actuators that has temporal dynamics similar to those for muscles.

Muscles are often modelled to act as second-order low-pass filters of the neural command (Zajac 1989) with chemical activation dynamics and mechanical force-generation (also called contraction) dynamics. Activation dynamics refers to the dynamics of calcium uptake/release in the muscle fibre once the neural spike train excites the tissue. Contraction dynamics refers to the dynamics of force generation in the muscle that depends on sarcomere mechanics and muscle plus tendon compliance. We developed a torque generation model with two time scales as proxies for muscle activation–contraction dynamics. The two time scales are one for ‘activation’ dynamics (*T*_act_ in equation (3.7*a*)) and another for ‘contraction’ dynamics (*T*_con_ in equation (3.7*b*)). We also include the well-known asymmetry in activation versus deactivation time scales (Brown & Loeb 2000) caused by the faster uptake of calcium compared to the release of calcium (parametrized by *β* in equations (3.7*a*)–(3.8)).

For each joint *i*=1, 2, 3, the differential equations for torque production are governed by a hidden internal variable that we will refer to as ‘activation’ and denote by the symbol $a^{\left(i\right)}(t)$:

(3.7a) $$T_{\text{act}}{\dot{a}}^{\left(i\right)}+\left(\beta +(1-\beta )\left|\tau_{\text{D}}^{\left(i\right)}\right|\right)a^{\left(i\right)}=\tau_{\text{D}}^{\left(i\right)},$$

(3.7b) $$T_{\text{con}}{\dot{\tau }}^{\left(i\right)}+\tau^{\left(i\right)}=a^{\left(i\right)}.$$

Time dependence has been omitted for clarity. Combining the above two equations, we obtain a second-order nonlinear ordinary differential equation model for torque production. The nonlinearity arises because of the dependence (governed by *β*) of the activation–deactivation time constant on the driving input command (***τ***_D_):

(3.8) $$T_{\text{act}}T_{\text{con}}{\ddot{\tau }}^{\left(i\right)}+\left(T_{\text{act}}+T_{\text{con}}\left(\beta +(1-\beta )\left|\tau_{\text{D}}^{\left(i\right)}\right|\right)\right){\dot{\tau }}^{\left(i\right)}+\left(\beta +(1-\beta )\left|\tau_{\text{D}}^{\left(i\right)}\right|\right)\tau^{\left(i\right)}=\tau_{\text{D}}^{\left(i\right)},$$

where ***τ***_D_ is the torque command signal that we want to find as part of the optimal control problem.

*Numerical values for model parameters*. We used values for *T*_act_, *T*_con_ and *β* based on calculations similar to those given in Zajac (1989). To allow for the fastest possible, but biologically plausible, torque actuators, we chose *T*_act_=12 ms based on existing experimental data on fast muscle fibres (Wells 1965; Close 1972; Zajac 1989). Note that this value is slightly faster than typical values reported for human muscles (e.g. Winters & Stark 1985). For calculating *T*_con_, note that the biggest muscles actuating the finger are located in the forearm and therefore possess long tendons (Lieber 1993). Using a calculation similar to Zajac (1989) we chose *T*_con_=3*T*_act_=36 ms. We chose *β*=0.2 based on the existing experimental data (Winters & Stark 1988). This is similar to values used by others for muscle models (e.g. Raasch *et al*. 1997).

### (e) Numerical optimization

For the numerical optimal control problem, we focus only on the time interval from −60 to 0 ms, where 0 ms is the contact time. We fully specify the initial condition of the finger $\left(\varphi_{\text{m}},{\dot{\varphi }}_{\text{m}},\tau_{\text{m}},{\dot{\tau }}_{\text{m}}\right)$ at −60 ms and find *τ*_D_ to minimize a cost function that depends on the finger's state at contact (***r***(0), ${\dot{r}}_{x}(0)$, ${\dot{r}}_{\alpha }(0)$, *τ*(0) and $\dot{\tau }(0)$). For numerical efficiency without compromising the time resolution of the joint torque dynamics, we time-discretize the torque command (***τ***_D_), using a piecewise linear approximation defined on a mesh of four points in the time interval [−60 ms, 0 ms]. Thus, the command varies linearly in pieces of 20 ms duration. A duration of 20 ms is faster than the rate-limiting time scale of joint torque dynamics, namely *T*_con_=36 ms. Moreover, this choice of time discretization is biologically reasonable based on the known peak discharge rates of spinal motor neurons. Neurophysiological studies report ranges for measured peak discharge rates between 35 and 45 Hz (Fuglevand *et al*. 1993). Modelling studies typically use values in the range of 25–50 Hz (Brown & Loeb 2000; Jones *et al*. 2002; Keenan & Valero-Cuevas 2007). We now outline the numerical optimization procedure.

#### (i) Calculating initial conditions

Resembling our experimental data (Venkadesan & Valero-Cuevas 2008), at *t*=−500 ms we specify the fingertip to be at rest, located vertically level with the MCP joint, vertically above the target, and with a vertically oriented distal phalanx. During the interval [−500 ms, −60 ms], we prescribe the fingertip to move downwards, with the distal phalanx remaining vertical, and the fingertip acceleration being a *β*-function such that the fingertip exactly reaches the surface height at *t*=0 ms. For this calculation, we also assume that the fingertip horizontal velocity and distal phalanx angular velocity are both zero. The corresponding fingertip velocity profile is a smooth, symmetric sigmoid. These conditions resemble our experimental data and uniquely specify the time series of the finger's state (***φ***, $\dot{\varphi }$, ***τ***, $\dot{\tau }$) until *t*=−60 ms. Thus, the conditions at *t*=−60 ms are specified.

#### (ii) Cost function

The cost function was calculated using the finger's state at *t*=0 ms, at the end of a dynamic simulation of finger motion during the interval [−60 ms, 0 ms] driven by the piecewise linear control command *τ*_D_(*t*):

(3.9) $$J={\left(\frac{r_{x}-L}{10}\right)}^{2}+{\left(\frac{r_{y}-H}{3}\right)}^{2}+{\left(\frac{r_{\alpha }-0}{\pi /10}\right)}^{2}+{\left(\frac{{\dot{r}}_{x}-0}{100}\right)}^{2}+{\left(\frac{{\dot{r}}_{\alpha }-0}{\pi /2}\right)}^{2}+{\left\|\frac{\tau (0)-k_{1}{\hat{\tau }}_{\text{f}}}{k_{1}}\right\|}^{2}+{\left(\frac{\text{arccos}(\hat{\dot{\tau }}\cdot {\hat{\tau }}_{\text{f}})-0}{\pi /18}\right)}^{2},$$

where *L* is the horizontal location of the target relative to the MCP joint (figure 3); *H* is the corresponding vertical location; *k*_1_ is the desired magnitude of the immediate post-contact joint torque vector; and ${\hat{\tau }}_{\text{f}}$ is the unit vector for joint torques that would produce a vertically oriented fingertip force in the desired contact posture (figure 3). Note that the sixth term is a sum over the three components of the joint torque vector at contact.

We divide the error in each component by a scaling factor to non-dimensionally define the term ‘small error’. The scaling factors for each term in equation (3.9) were chosen so that the cost function is negligibly small if and only if at *t*=0 ms: Δ*r*_*x*_≪10 mm; Δ*r*_*y*_≪3 mm; Δ*r*_*α*_≪*π*/10 rad; $\Delta {\dot{r}}_{x}\ll 100\;\text{mm}\;{\text{s}}^{-\text{1}}$; $\Delta {\dot{r}}_{\alpha }\ll \pi /2\;\text{rad}\;{\text{s}}^{-1}$; $\Delta \tau^{\left(i\right)}\ll k_{1}\;\text{N}\;\text{m}$ for *i*=1, 2, 3; and $\text{arccos}(\hat{\dot{\tau }}\cdot {\hat{\tau }}_{\text{f}})\ll \pi /18$ rad. Note that larger errors in this cost function also mean larger deviation of the fingertip force vector direction from vertical, because ${\hat{\tau }}_{\text{f}}$ was calculated to produce vertical force at the specific target posture and zero joint angular velocities. Therefore, equation (3.9) serves as a surrogate for the actual cost we are interested in.

#### (iii) Initial guess

For generating the initial guess, we first extend the finger joint-angle trajectories obtained in §3*e*(i) to the interval [−500 ms, 0 ms]. We then solve for ***τ***(*t*) by substituting for ***φ***(*t*), $\dot{\varphi }(t)$ and $\ddot{\varphi }(t)$ in equation (3.1) and find $\dot{\tau }(t)$ by differentiating ***τ***(*t*). By substituting ***τ***(*t*) and $\dot{\tau }(t)$ in equation (3.8) we form ***τ***_D_(*t*). Thus, we form the initial guess of *τ*_D_ at four uniformly spaced mesh points of the interval [−60 ms, 0 ms].

#### (iv) Optimization loop

We first make an initial guess for ***τ***_D_ as outlined in §3*e*(iii) and use the function fminsearch within the Matlab simulation environment to find the optimal control solution. This optimization routine uses the Nelder–Mead simplex method. Despite unprovable convergence (Lagarias *et al*. 1998) in problems with more than two dimensions (our problem is 4×3=12 dimensional), we chose this algorithm because it does not use gradient information and is hence robust. Moreover, we performed additional steps to verify convergence as outlined below. Other smooth, gradient-based methods failed to converge for our problem, and stochastic methods such as simulated annealing were too slow to converge.

In each iteration of fminsearch, we solve the differential equations (3.1) and (3.8) in the interval [−60 ms, 0 ms] using initial conditions specified in §3*e*(i). We use the values of ***φ***(0), $\dot{\varphi }(0)$, ***τ***(0) and $\dot{\tau }(0)$ at the termination of the dynamic simulation of finger motion and equation (3.9) to calculate the cost function.

#### (v) Convergence criteria

We consider a solution to have converged if and only if all of the following criteria are satisfied. First, we require the solution to remain unchanged (to a tolerance of 10^−10^) for at least 5000 iterations of the optimization loop. Once that condition is satisfied, we then randomly perturb the solution using a random value drawn from a uniform distribution with a range of ±1 per cent of the solution value. Finally, using this perturbed solution as an initial guess, we verify whether the optimization converges back to the unperturbed solution (using the same tolerance of 10^−10^) before calling it a locally optimal solution. We repeated the random perturbation three times for each solution, all of which converged back to the same solution.

## 4. Results and discussion

We solved a sequence of optimization problems with increasing values of *k*_1_ (magnitude of the joint torque vector upon contact) from 0.015 to 0.1 N m. This corresponded to larger force magnitudes at contact, i.e. with increasing requirements on initial fingertip force magnitude from low (0.385 N) to high (1.889 N). All of these simulations converged successfully as per our criteria. Specifically, in all cases, the converged solution had a ‘perfect’ residual cost that was less than or equal to 10^−6^ (equation (3.9)). Individual terms in the cost function were each less than 10^−6^. This means that the error with respect to desired accuracy for every term in the cost function was less than 0.1 per cent. The corresponding angular deviation of the fingertip force vector from the vertical was always less than 10^−3^ rad. Our results were also robust to variability in the initial guess, although convergence to the final solution was faster if we used numerical continuation for finding the optimal solution for increasing values of *k*_1_. As an example of numerical continuation, say we have a converged solution for *k*_1_=0.015 N m and want to solve for *k*_1_=0.02 N m. Then, we would first solve for *k*_1_=0.016 N m using the converged solution for *k*_1_=0.015 N m as the initial guess. We repeat this slow increase in *k*_1_ until we reach *k*_1_=0.02 N m. This process is referred to as numerical continuation and is effective when there is a smooth (at least continuous) dependence of the output on a varying parameter, which in our problem is the dependence of optimal ***τ***_D_ on *k*_1_.

### (a) Agreement with experimental data

#### (i) Control switching: joint torque direction before magnitude

**Figure 4:**
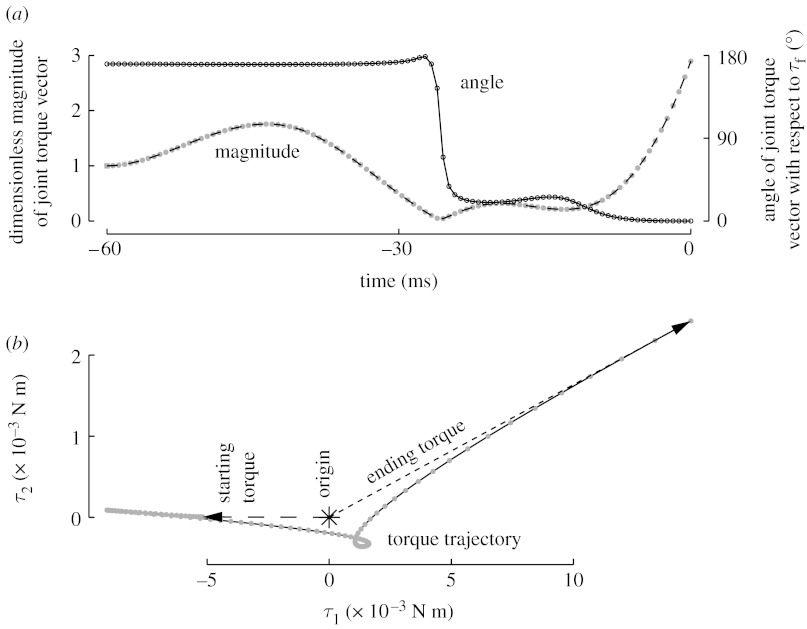
Optimal joint torque transitions for a low demand on post-contact joint torque magnitude (0.015 N m). For this low level of initial force production (0.385 N), the optimal controller found a solution that produces perfectly vertically aligned fingertip force upon contact. We show 100 uniformly spaced data markers over 60 ms. (*a*) The magnitude and direction of the joint torque vector. The angle between the joint torque vector before contact and the torque needed for maximal force production anticipatorily switched at approximately 30 ms before contact. The magnitude of the joint torque vector, however, increases only after reorientation of the joint torque vector. This closely resembles our experimental data. (*b*) A three-dimensional plot of the joint torque trajectory reveals why the torque vector direction changes before the magnitude. The joint torque vector for producing motion first shrinks in magnitude to close to the origin, then it reorients towards the torque vector needed for force production, and finally it increases in magnitude. Note that the three-dimensional plot looks planar (*τ*_1_–*τ*_2_ plane) because *τ*_3_(*t*)=0 for all of our optimal solutions.

As in the experimental data, figure 4 clearly shows that (i) changes in the joint torque vector direction are completed at approximately 30 ms before contact and (ii) growth in vector magnitude begins in earnest only after the change in vector direction is completed. This is in contrast to the other possible alternatives where torque vector magnitude and direction change simultaneously, or changes in direction lag changes in magnitude.

We present physical arguments for why the optimal solution shows this sequencing of change in vector direction followed by vector magnitude increase. The finger is very light and requires low torques for producing the desired motion. Therefore, if the magnitude of the torque vector increased rapidly while the finger is far from contact and joint torques are still transitioning, these high torques may overaccelerate the fingertip away from the target or cause the finger to land in an undesirable posture. In a real biological finger it would also make it necessary to dissipate more energy at impact, or have other possible undesirable consequences (e.g. slipping, bouncing, etc.). Thus, it is best to maintain low torque magnitudes for as long as possible.

#### (ii) Anticipatory nature of control switching

With respect to timing, our simulations are meant to shed light on our experimental results and therefore explore only the space of anticipatory joint torque strategies. However, the specific timing of direction change in the optimal solution was surprising. Given how rapidly the vector direction changed (less than 10 ms), the controller could have waited to execute the switch until just before contact (say, until *t*=−10 ms). Instead, the switch was executed well before contact (approx. 30 ms), in agreement with the experimental data. Although it is tempting to attribute the early switch in the biological system to uncertainties and noise, that is not applicable to our results. Our optimal controller had exact knowledge of the contact time and there was no stochastic element in our simulations.

We provide some physical arguments for this result. In §4*a*(i), we presented physical arguments for the sequencing of direction change followed by magnitude increase in the joint torque vector. However, for small values of ***τ***_D_, i.e. when the joint torque vector is close to the origin, the actual time scale of the contraction dynamics is slower than *T*_con_=36 ms because of the nonlinearity introduced by *β* (equation (3.8)). Therefore, the time taken to increase joint torques might be so severely rate limited that the magnitude increase has to start well in advance, and, in turn, the direction change has to be earlier still. In other words, given that a specific force magnitude is required at contact, the controller does indeed wait until the last minute to perform the transition, but the rate-limiting process is the magnitude increase and not the direction change. Note that we used smaller than typical values for *T*_act_ and *T*_con_ in our model (§3*d*) to truly estimate the upper bounds on the physical limits of performance. In the biological system, these time scales are likely to be slower and a real finger has additional joint viscoelastic elements that are absent in the model, which might explain the approximate 35 ms discrepancy in exact timing of the switch between our model and the experimental results.

### (b) Speed–accuracy trade-off

**Figure 5:**
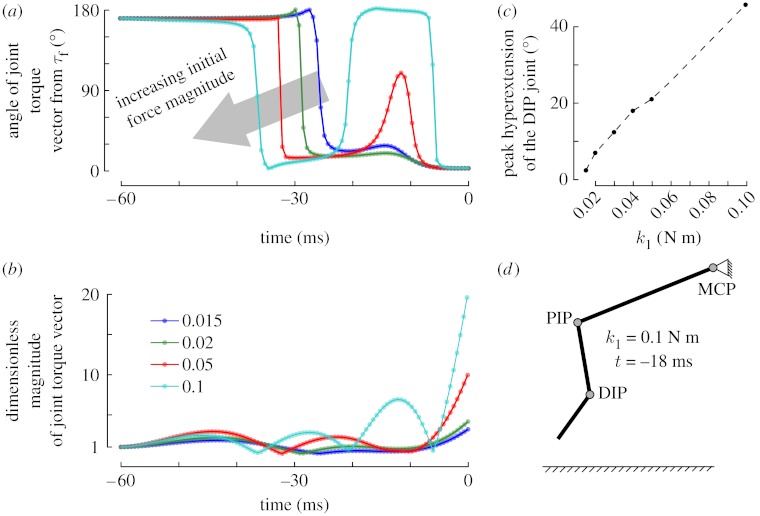
Transition of the joint torque vectors for increasing demands on the initial force magnitude (what we call ‘speed’). Despite a 20× range in initial fingertip force magnitude (6.6×range in *k*_1_), a perfectly accurate solution was always found, i.e. the fingertip force vector was perfectly vertically oriented upon contact and produced the required force magnitude. The orientation of the joint torque vectors and the joint angles during the transition provide some insights into this result. (*a*) Orientation of the joint torque vector has a ‘double dip’ for higher speed demands (*k*_1_≥0.05), something akin to a ‘corrective’ torque to optimize the finger's state upon contact. Note that this corrective action occurs even though our model has no sensory feedback. (*b*) Magnitude increases only after reorientation of the joint torque vector. (*c*) These corrective torques, however, were associated with intermediate postures that were unrealistically hyperextended at the DIP joint. As seen here, the magnitude of an unrealistic hyperextension of the DIP joint grows almost linearly with increasing speed demands, i.e. increasing *k*_1_. (*d*) For *k*_1_=0.1 N m, the figure shows the unrealistic finger posture attained at *t*=−18 ms. We show only four of six values of *k*_1_ in (*a*) and (*b*) for clarity.

As the demands of the task increase, which in our problem is the magnitude of the immediate post-contact fingertip forces (represented by *k*_1_), we see that the control switching happens earlier. More interestingly, when we compare increasing values of *k*_1_ from 0.015 to 0.1 N m, we see a qualitative difference emerge in the optimal control strategy (figure 5). Namely, the joint torque vector reorients itself twice for higher values of *k*_1_, as opposed to only once for lower values of *k*_1_.

Upon further inspection of the more demanding tasks, we find that the solutions with corrective torques also exhibit unrealistically hyperextended DIP joint angles in their motion (e.g. finger snapshot shown in figure 5*d* at approx. −18 ms for *k*_1_=0.1 N m). This anatomically unrealistic action is what helps the optimal controller to achieve a perfect solution (residual is approx. 0) for even the demanding tasks. This is an artefact of insufficient constraints in our model because we do not impose restrictions on the range of motion of the joints.

Our proposition is that if we were to solve the (harder to converge) constrained optimization problem by adding joint constraints, the optimal controller would no longer be able to execute perfect transitions. In other words, we expect that, with the addition of joint constraints, the error in fingertip force vector direction would be greater for increasing demands on initial force magnitude. This proposition is supported by figure 5, where we plot the peak hyperextension of the DIP joint (i.e. the peak value of unrealistic postures) as a function of *k*_1_. It is apparent that there is a nearly linear and monotonic increase in the unrealistic hyperextension of the DIP joint with increasing values of *k*_1_. Given that this was the unique optimal solution with a large basin of attraction (because perturbations of this solution converged back to it), constraining the controller to avoid joint hyperextension would only increase the residual cost function, i.e. there would be greater errors in fingertip force vector direction for higher demands on the speed of rise of force magnitude. This is because, in the neighbourhood of the minimum, the cost function resembles a quadratic surface. Therefore, by excluding increasingly larger neighbourhoods of the optimal solution (for increasing constraint violations), we expect the cost function to increase monotonically (in the absence of nearby minima).

Our speed–accuracy result remains inconclusive until we solve the constrained optimization problem. But we are confident that future refinements to the model will reveal this trend of a speed–accuracy trade-off. We conclude by hypothesizing that, with higher demands on the initial speed of force rise, the transition has to start sooner because of limitations imposed by neuromuscular lags, thus causing greater errors in the fingertip force vector direction upon contact. This hypothesis is already indirectly supported by experimental data showing that healthy subjects cannot perform the transition without incurring error in the initial force direction (Venkadesan & Valero-Cuevas 2008), and a report showing that reducing the friction of the target surface advances the time of the transition (Medina *et al*. 2007).

A further comment on the prevailing theories of speed–accuracy trade-off is warranted. Starting with the paper by Paul Fitts, noise in the sensorimotor system has been implicated as the origin of speed–accuracy trade-offs in human behaviour (Fitts 1954). With the discovery of signal-dependent noise in muscles, most contemporary research considers noise as the reason for speed–accuracy trade-offs (Meyer *et al*. 1990; Harris & Wolpert 1998, 2006; Todorov 2004, 2005). However, there was an alternative theory proposed by Card *et al*. (1983) explaining speed–accuracy trade-offs based on time delays in deterministic systems. This theory has been challenged by many on the basis of unrealistic assumptions, such as the existence of ‘submovements’ for any movement, etc. (see Meyer *et al*. (1990, pp. 192–3) for a detailed critique of Card *et al*.'s theory). A contribution of our work is, therefore, to discover speed–accuracy trade-offs in the context of deterministic control transitions with physiologically plausible neuromuscular lags. At the very least, our model's findings complement and serve as an alternative to prevailing noise-based theories. At best, it proposes a deterministic route to speed–accuracy trade-offs that occur for the ubiquitous, yet critical, task of transitioning between control regimes.

### (c) Near-optimal performance by humans

The physical bounds of performance identified by our simple model of the finger robustly reproduce experimental observations made on the real human despite excluding some known physiological properties and other simplifications. Our results therefore suggest that our model implements some fundamental mechanism whose importance overrides the simplifying assumptions. A possible alternative result could have been that our solutions always converged to large residual costs (or did not converge at all) because requiring torques to transition before contact is simply mechanically detrimental. By contrast, we find that our idealized model was able to arrive at perfect solutions whose predicted torque time histories replicate the main features of the biological data. In addition, the details of the torque transitions are sensitive to changes in the desired initial force magnitude, which argues for a gradient in performance for which some form of optimization is beneficial. These results lead us to conclude that the behaviour of the nervous system is indeed near-optimal. Even if the reader disagrees that the similarity of the predictions with the experimental data implies near-optimality of human performance, it is clear that the resemblance suggests, at the very least, that the transitions in control command in this context are mostly governed by mechanical principles and requirements (as opposed to being dominated by non-mechanical neural/behavioural/idiosyncratic constraints) and indeed approach the physical boundaries of performance. This conclusion agrees well with, and extends into, the dynamical domain, prior work showing that the control of finger musculature for force production is mostly governed by mechanical principles (Valero-Cuevas *et al*. 1998).

### (d) Scope and limitations

We have presented here two main results. (i) The experimental observations of anticipatory switching of joint torques and the sequencing of the joint torque vector's angle change followed by magnitude can both be explained solely by neuromuscular lags in the context of control switching. (ii) Our modelling suggests that, when switching between mutually incompatible control regimes, speed–accuracy trade-offs arise solely from neuromuscular lags. This is an alternative explanation to many current theories that require stochastic elements in their models for speed–accuracy trade-offs.

While our results (figure 5) support the hypothesis that speed–accuracy trade-offs can arise solely from neuromuscular lags, we do not have a definitive proof. A definitive proof would require solving a constrained optimization and verification that the solution is a unique global optimum. While a constrained optimization is something we will pursue in the future, guaranteeing that the solution is a unique global optimum probably requires analytical proofs (not just numerical demonstration), which is beyond the scope of this first numerical exploration of biomechanical finger contact transitions. However, all the solutions we found made the cost function vanish, i.e. there are no other solutions with a lower value of the cost function. Therefore, we can guarantee that all solutions were globally optimal (i.e. smallest possible cost, though possibly non-unique) and probably non-degenerate.
